# Requirement of ABC transporter inhibition and Hoechst 33342 dye deprivation for the assessment of side population-defined C6 glioma stem cell metabolism using fluorescent probes

**DOI:** 10.1186/s12885-016-2895-8

**Published:** 2016-11-04

**Authors:** Yoshitaka Murota, Kouichi Tabu, Tetsuya Taga

**Affiliations:** Department of Stem Cell Regulation, Medical Research Institute, Tokyo Medical and Dental University (TMDU), Bunkyo-ku, Tokyo, 1138510 Japan

**Keywords:** Cancer stem cells, Side population, Fluorescent probes, ABC transporters, JC-1, Hoechst 33342

## Abstract

**Background:**

Elucidating the precise properties of cancer stem cells (CSCs) is indispensable for the development of effective therapies against tumors, because CSCs are key drivers of tumor development, metastasis and relapse. We previously reported that the Hoechst 33342 dye-low staining side population (SP) method can enrich for CSCs in the C6 glioma cell line, and that the positively stained main population (MP) cells are non-CSCs. Presence of cancer stem-like SP cells is reported in various types of cancer. Although altered cellular energy metabolism is a hallmark of cancer, very little has been studied on the applicability of fluorescent probes for the understanding of CSC energy metabolism.

**Methods:**

The metabolic status of C6 SP and MP cells are evaluated by CellROX, MitoTracker Green (MTG) and JC-1 for cellular oxidative stress, mitochondrial amount, and mitochondrial membrane potential, respectively.

**Results:**

SP cells were found to exhibit significantly lower fluorescent intensities of CellROX and MTG than MP cells. However, inhibition of ATP binding cassette (ABC) transporters by verapamil enhanced the intensities of these probes in SP cells to the levels similar to those in MP cells, indicating that SP cells expel the probes outside of the cells through ABC transporters. Next, SP cells were stained with JC-1 dye which exhibits membrane potential dependent accumulation in mitochondrial matrix, followed by formation of aggregates. The mitochondrial membrane potential indicated by the aggregates of JC-1 was 5.0-fold lower in SP cells than MP cells. Inhibition of ABC transporters enhanced the fluorescent intensities of the JC-1 aggregates in both SP and MP cells, the former of which was still 2.2-fold lower than the latter. This higher JC-1 signal in MP cells was further found to be due to the Hoechst 33342 dye existing in MP cells. When SP and MP cells were recultured to deprive the intracellular Hoechst 33342 dye and then stained with JC-1 in the presence of verapamil, the intensities of JC-1 aggregates in such SP and MP cells became comparable.

**Conclusion:**

Inhibiting ABC transporters and depriving Hoechst 33342 dye are required for the accurate assessment of side population-defined C6 glioma stem cell metabolism using fluorescent probes.

## Background

Metabolic alteration in cancer cells has been considered to be linked to cancer progression and drug resistance, and thus characterized intensively for a long time as a potential therapeutic target [1]. In addition, the evidence has been accumulating that tumors contain a small subset of stem-like cells called cancer stem cells (CSCs), a key driver of tumor development, metastasis and relapse [2]. As CSCs among bulk cancer cells are resistant to conventional chemo/radio-therapies and have critical roles in recurrence, elucidation of their unique aspects of metabolism is therefore expected to provide us clues to develop novel therapeutic strategies against tumors. To date, several researchers have investigated cellular energy metabolism of CSCs; Zhou et al. reported that the ATP generation in CSCs is dependent on glycolysis [3]. On the other hand, Vlashi et al. reported that the CSC ATP generation mainly relies on oxidative phosphorylation [4]. Because the characteristics of CSC metabolism are still controversial, it is imperative to accumulate more evidence in more accurate ways. Previously, we reported that the Hoechst 33342 dye-effluxing SP cells, but not the dye-retaining MP cells of the C6 glioma cell line are rich in CSCs with such properties as high tumorigenicity [5]. SP-defined CSCs have been reported in various types of cells [6–10]. The high dye-effluxing activity of SP cells is known to be dependent on ATP binding cassette (ABC) transporters, which are also highly expressed in a variety of normal tissue stem cells [11].

Fluorescent probes have been widely used for the evaluation of cellular metabolic status such as intracellular reactive oxygen species (ROS) levels, mitochondrial amount and mitochondrial membrane potential [12]. CellROX Deep Red Reagent (CellROX) is used to determine cellular ROS levels, which is non-fluorescent in the reduced state and becomes fluorescent (excitation/emission maxima of 640/665 nm) when oxidized by intracellular ROS [13]. MitoTracker Green (MTG) (excitation/emission maxima of 490/516 nm) is a carbocyanine-based dye widely used to evaluate the amount of mitochondria. MTG labels mitochondria reportedly via the interaction with anionic phospholipid cardiolipine, which is abundant in the inner mitochondrial membrane [14]. JC-1 is a lipophilic cationic dye for the evaluation of mitochondrial membrane potential. The maximum excitation wavelength of JC-1 monomers is 498 nm and that of JC-1 aggregates is 593 nm. JC-1 molecules exist as a monomer at low concentrations. When loaded into the mitochondrial matrix upon membrane potential-driven uptake, JC-1 molecules are concentrated and form aggregates, which lead to the shift of the maximum emission wavelength from 525 to 595 nm [15].

While extensive research has been done on the high activity of CSCs to expel xenobiotic compounds outside of the cells through ABC transporters, the applicability of fluorescent probes for assessing the CSC metabolism has not been sufficiently studied. In fact, some fluorescent probes are already known as substrates of ABC transporters [16–18], but a detailed examination of the appropriateness of their use for CSC research has not been made. In this study, with the use of C6 glioma SP cells as an established CSC model, we examined three fluorescent probes, CellROX, MTG and JC-1 for their applicability to the assessment of SP-defined CSC metabolism, and precisely compared the metabolic differences between CSCs and non-CSCs.

## Methods

### Cell culture and SP analysis

C6 cells were maintained under 5 % CO_2_ at 37 ˚C in Dulbecco’s modified Eagle’s medium (DMEM, Wako) supplemented with 10 % fetal bovine serum (FBS) and 50 U/ml penicillin and 50 μg/ml streptomycin (Gibco). Detection and isolation of SP cells were performed as previously described [5]. Briefly, C6 cells were harvested by trypsinization, resuspended in 2 % FBS-DMEM at a final density of 1 × 10^6^ cells/ml, and incubated in dark with 5 μg/ml Hoechst 33342 dye for 90 min at 37 ˚C in the presence or absence of 50 μM verapamil. One μg/ml propidium iodide was used to discriminate dead cells. SP and MP cells were separated by FACS Aria II (BD Biosciences) equipped with 355 nm UV laser. The fluorescence of Hoechst 33342 dye was monitored through 405/20 nm band pass filter and 670 nm long pass filter.

### CellROX, MTG and JC-1 staining

Three fluorescent probes were purchased from Molecular Probes, dissolved in dimethylsulfoxide (DMSO) (100 mM, 500 μM, and 1 mg/ml stock concentrations, respectively), and stored at −20 ˚C in dark. Experimental conditions were indicated as follows: cells were resuspended in 2 % FBS-DMEM at a density of 1 × 10^5^ cells/ml and incubated at 37 ˚C with 5 μM CellROX for 30 min, 100 nM MTG for 30 min, or 1 μg/ml JC-1 for 40 min. The fluorescent probes were excited with 488 nm (for MTG and JC-1) or 633 nm (for CellROX) laser, and monitored through 530/30 nm band pass (for MTG and JC-1 monomers), 575/25 nm band pass (for JC-1 aggregates) or 660/20 nm band pass (for CellROX) filter. The mean fluorescent intensity (MFI) was calculated using FlowJo software version 7.6.5 (TOMY Digital Biology).

### Statistical analysis

All data are shown as the means ± standard deviation (SD) from three independent experiments. Significance between experimental groups was determined by Student’s t-test. A two-tailed value of less than 0.05 was considered significant.

## Results

### CellROX, MTG and JC-1 are excluded from SP-defined CSCs through ABC transporters

**Fig. 1:**
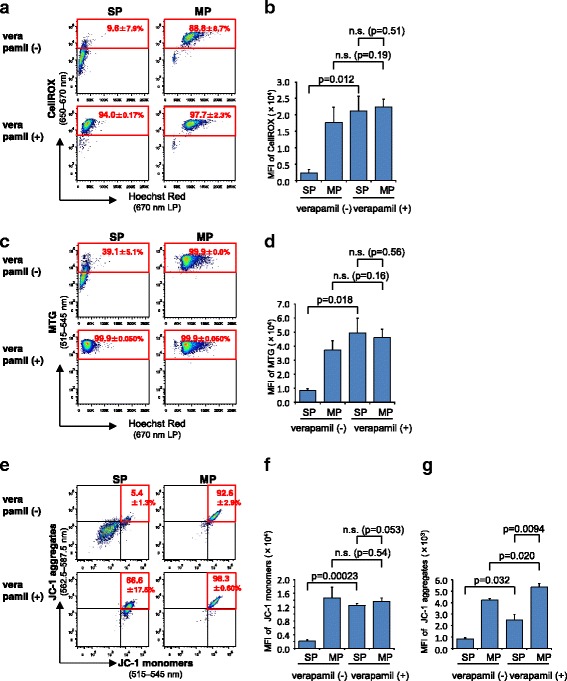
The influence of ABC transporters on the fluorescence of probes in SP and MP cells. FACS plots and mean fluorescent intensity (MFI) of sorted C6 SP (Hoechst 33342-effluxing) and MP (Hoechst 33342-retaining) cells after staining with CellROX (**a**, **b**), MTG (**c**, **d**) and JC-1 (**e**-**g**) in the presence or absence of verapamil. Fluorescence of each probe was analyzed using the indicated filters. The mean percentages ± SD of positive cells per total cells are shown in plots. n.s., not significant. *n* = 3

To examine whether fluorescent probes are applicable to the assessment of CSC metabolism, we first sorted SP and MP cells (defined as CSCs and non-CSCs, respectively) from the C6 glioma cell line by FACS. The sorted cells were subsequently stained with three fluorescent probes, CellROX (for ROS levels), MTG (for mitochondrial amount) or JC-1 (for mitochondrial membrane potential). To see the effect of ABC transporters known to be present at higher levels in SP cells than MP cells and expel the Hoechst 33342 dye, the staining process was done either in the presence or absence of 50 μM verapamil, an inhibitor of ABC transporters. The exclusion of Hoechst 33342 dye from C6 cells was negligible at this concentration of verapamil (data not shown). The treatment of verapamil significantly enhanced the CellROX fluorescence in SP cells, whereas the fluorescence of CellROX in MP cells was not affected (Fig. 1a and b), indicating that CellROX was excluded from SP cells through ABC transporters and that oxidative stress as measured by ROS formation in SP and MP cells was comparable. Similar results were obtained from experiments using MTG, which indicates that the mitochondrial amount is comparable in SP and MP cells (Fig. 1c and d). The mean fluorescent intensity (MFI) of JC-1 monomers and aggregates were 6.9-fold and 5.0-fold respectively lower in SP cells than MP cells without verapamil (Fig. 1e, f, and g). The MFI of JC-1 monomers in SP cells was significantly increased by verapamil treatment and C6 SP and MP cells displayed similar levels of fluorescent intensities of JC-1 monomers in the presence of verapamil (Fig. 1f, the third and fourth columns), which is comparable to that in MP cells without verapamil (Fig. 1f, the second column). The MFI of JC-1 aggregates in SP cells was significantly increased (by 193 %) by verapamil, and the MFI of JC-1 aggregates of MP cells was also increased to a lesser extent (by 26 %) (Fig. 1g). The MFI of JC-1 aggregates in SP cells was 46 % of that in MP cells in the presence of verapamil, whereas that in SP cells, in the absence of verapamil, was only 20 % of that in MP cells (Fig. 1g). These data indicate that the three metabolic indicators CellROX, MTG and JC-1 are excluded from SP cells through ABC transporters, and thus the influence of ABC transporters must be considered for the fluorescent probe-based assessments of the metabolic status of SP-defined CSCs. Considering the lower level of JC-1 aggregates in SP cells in the presence of verapamil (Fig. 1g), C6 glioma CSCs may seem to have lower mitochondrial membrane potential than non-CSCs.

### Hoechst 33342 dye prestaining enhances the fluorescent intensities of JC-1 aggregates

**Fig. 2:**
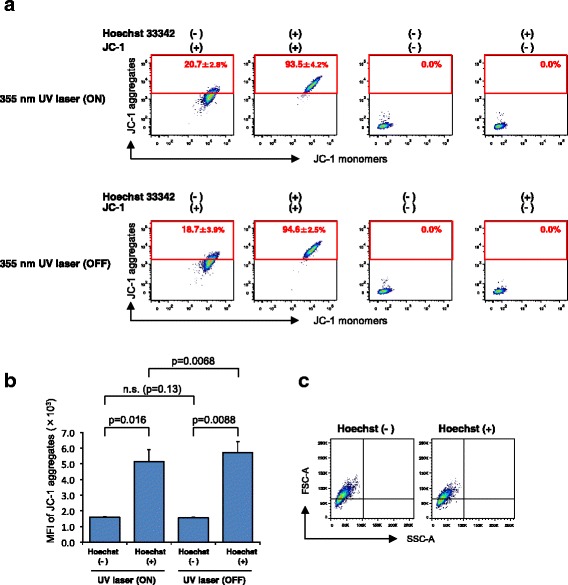
The influence of prestaining with Hoechst 33342 dye on the MFI of JC-1 aggregates. **a** FACS plots of C6 glioma cells stained with Hoechst 33342 dye and subsequently with JC-1 in the presence of verapamil. The upper and lower panels represent data from cells analyzed with or without Hoechst 33342-exciting 355 nm UV laser, respectively. The mean percentages ± SD of positive cells per total cells are shown in the plots. **b** Bar graph representing the mean fluorescent intensity of JC-1 aggregates of the JC-1-stained cells in (**a**). *n* = 3. **c** FSC/SSC plots of C6 glioma cells stained with (+) or without (−) Hoechst 33342 dye

Because the amounts of Hoechst 33342 dye in sorted SP and MP cells are different, with the latter being very high, the influence of Hoechst 33342 dye on the fluorescent intensity of JC-1 aggregates was further examined. As shown in Fig. 2a (upper panels), C6 cells stained with Hoechst 33342 dye displayed significantly higher levels of JC-1 aggregates. The difference was approximately 3.2 fold (Fig. 2b, left two columns). To explain this undesirable effect of Hoechst 33342 dye, we tested three hypotheses; 1) 355 nm UV laser for Hoechst 33342 might unexpectedly excite JC-1 aggregates, 2) fluorescence emitted from the UV-excited Hoechst 33342 might excite JC-1 aggregates, and 3) 488 nm argon laser for JC-1 excitation might unexpectedly excite Hoechst 33342 dye. However, even when UV laser was shut off, the fluorescent intensity of JC-1 aggregates in cells never decreased regardless of the staining without or with Hoechst 33342 and the MFI in the latter was always high (Fig. 2a, left panels and b). It is unknown why MFI of JC-1 aggregates is somewhat enhanced (by 11 %) following the shut off of the UV laser (Fig. 2b, second and fourth columns), but at least it is obvious that 355 nm UV laser does not excite JC-1 aggregates and UV laser-excited Hoechst 33342 does not excite JC-1 aggregates. When C6 cells were stained with Hoechst 33342 dye alone and excited by the argon laser, the emitted fluorescence from the cells is negligible through the filter for JC-1 aggregates (Fig. 2a, right panels). Finally, a possible influence of Hoechst 33342 staining on cell size and granularity, which might affect JC-1 aggregation, was examined. As shown in Fig. 2c, staining with Hoechst 33342 dye did not affect the size and granularity of cells (Fig. 2c). Therefore, we cannot yet figure out how Hoechst 33342 dye influences the fluorescence of JC-1 aggregates, but at least it is obvious that Hoechst 33342 staining interferes with the assessment of mitochondrial membrane potential using JC-1.

### Fluorescent intensity of JC-1 aggregates in SP and MP cells becomes comparable by depriving Hoechst 33342 dye and inhibiting ABC transporters

**Fig. 3:**
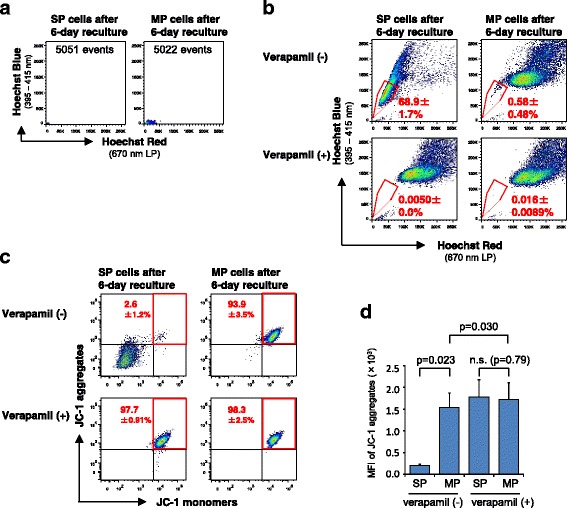
MFI of JC-1 becomes comparable when ABC transporters are inhibited and Hoechst dye is deprived. **a** Removal of intracellular Hoechst 33342 dye by reculturing SP and MP cells. Twenty thousands of cells were sorted by FACS and recultured for 6 days. **b** FACS plots of SP- and MP-derived cells restainined with Hoechst 33342 dye. The mean percentages ± SD of SP-derived SP cells (left panel) or MP-derived SP cells (right panels) detected on day 6 of reculture to derive initial Hoechst 33342 were indicated in the plots. **c** FACS plots of SP- and MP-derived cells stained with JC-1 in the presence or absence of verapamil. **d** The mean fluorescent intensity of JC-1 aggregates was calculated and shown as bar graphs. n.s., not significant. *n* = 3

To achieve the accurate assessment of the mitochondrial membrane potential using the JC-1 dye, sorted SP and MP cells were recultured for 6 days to deprive intracellular Hoechst 33342 dye and subjected to staining with JC-1. These cells were confirmed to possess no or negligible Hoechst 33342 dye (Fig. 3a). The recultured SP and MP cells were then stained with Hoechst 33342 again to know SP/MP profiles of these cultured cells. As shown in Fig. 3b, 68.9 ± 1.7 % of SP-derived cells retained the SP phenotype, and MP-derived cells displaying SP phenotype were negligible (0.58 ± 0.48 %). As shown in Fig. 3c (upper panels) and d, the recultured MP cells deprived of initial Hoechst 33342 dye displayed higher levels of fluorescence whose wave length corresponds to JC-1 aggregates than SP cells in the absence of verapamil, as expected. However, SP- and MP-derived recultured cells exhibited similar levels of fluorescence corresponding to JC-1 aggregates in the presence of verapamil (Fig. 3c lower panels and d), suggesting that mitochondrial membrane potential of C6 CSCs are comparable to that of C6 non-CSCs. Taken together, our data indicate that the activity of ABC transporters and the presence of Hoechst 33342 dye in cells definitely interfere with the estimation of the metabolic status when fluorescent probes are applied to SP-defined CSCs.

## Discussion

Understanding of the metabolic status of CSCs is considered to provide clues for the development of eradicative therapies against tumors. In the present study, we assessed whether three widely used fluorescent probes CellROX, MTG and JC-1 are applicable to the study of metabolic differences between CSCs and non-CSCs from the C6 glioma cell line. We here demonstrate that 1) SP-defined rat C6 glioma CSCs expel these fluorescent probes outside of the cells through ABC transporters, and 2) staining with the Hoechst 33342 dye used for SP/MP separation, in other words, identification process of C6 glioma CSCs and non-CSCs, enhances the fluorescent intensity of JC-1 aggregates. Thereby, we propose that inhibition of probe-exclusion is required for the comparison of the metabolic status in SP-defined C6 glioma CSCs and non-CSCs with the use of fluorescent probes. In addition, deprivation of the Hoechst 33342 dye used for C6 glioma CSC/non-CSC identification is required for the accurate assessment of the mitochondrial membrane potential using JC-1.

CSCs can be isolated based on their characteristics such as the surface expression of stem cell markers like CD133 or CD44 and low 26S proteasome activity [19]. In addition, the SP method based on the activity of ABC transporters which is strongly associated with anti-cancer drug-resistant properties of CSCs is widely utilized to enrich CSCs in many types of tumors [6–10, 20]. Our results therefore generally provide an important caution that we may easily misestimate the metabolic status of CSCs and non-CSCs when fluorescent probes are used. In fact, some investigators actually compare the metabolic status of CSCs and non-CSCs using fluorescent probes without considering the possibility that CSCs expel such probes [21–24].

MTG and JC-1 have already been demonstrated as substrates of ABCB1, ABCC1 and ABCG2 [16–18]. Our previous cDNA microarray data for the expression of some ABC transporter genes (available in NCBI GEO repository #GSE72431) reveal that only ABCB1 gene is specifically upregulated in C6 glioma SP cells compared with MP cells approximately by 10.6-fold (SP:MP = 0.64:0.060), suggesting that the ability of SP cells to expel xenobiotics is mainly dependent on ABCB1 in C6 cells [25, 26]. In addition, it has been reported that ABCB1 is overexpressed in human glioma tissues and its expression level is strongly correlated with that of another glioma CSC marker CD133, suggesting the importance of ABCB1 in glioma CSCs [27]. We also show here that CellROX is a newly identified substrate of verapamil-targeted ABC transporters, as well as that MTG and JC-1 are substrates of ABC transporters in glioma.

In Figs. 1g and 3d, we observed a slight enhancement of JC-1 aggregates, but not JC-1 monomers, in MP cells treated with verapamil despite the fact that the MFI of CellROX and MTG in these cells were not affected by this treatment. Concerning this phenomenon, verapamil might inhibit the ABC transporters barely existing in the mitochondrial inner membrane which may possibly regulate the concentration of JC-1 in mitochondrial inner membrane, eventually affecting the formation of red aggregates. However, the presence of ABC transporters in mitochondrial inner membrane and effects of verapamil to them in C6 glioma cells should be further investigated. In any case, the increase in MFI of the JC-1 aggregates in MP cells treated with verapamil was much lower compared with that observed in SP cells.

As for the mechanisms underlying the enhancement of the fluorescent signal corresponding to JC-1 aggregates by Hoechst prestaining, it is possible that colocalization of JC-1 molecules with the dye in the mitochondrial matrix or mitochondrial DNA damage induced by Hoechst staining [28] might change the fluorescent properties of JC-1 aggregates or the aggregating efficiency of JC-1 monomers in mitochondria. One paper reported that Hoechst 33342 dye opposingly decreases mitochondrial membrane potential measured by JC-1 in the myelogenous leukemia cell line HL-60 [29]. The influence of Hoechst 33342 dye might differ depending on the tissue types of tumor origin or subpopulations within tumor cells. In any case, therefore, attention should be paid to the effect of Hoechst 33342 on the fluorescence of JC-1 aggregates.

Altogether, establishing the accurate methods to precisely characterize CSCs will help us identifying ideal targets and develop eradicative therapies against cancer, though it is indispensable to further consider the influence of *in vivo* microenvironment such as hypoxia, low nutrients, and inflammation for thorough elucidation of the complex properties of CSC metabolism.

## Conclusion

We provide important cautions for the fluorescent probe-based assessments of cellular metabolism in C6 glioma CSCs isolated by the SP method, i.e. requirement of ABC transporter inhibition and Hoechst 33342 dye deprivation, by demonstrating the ability of glioma SP cells to expel fluorescent probes and the unexpected effect of Hoechst 33342 on the fluorescence corresponding to JC-1 aggregates. This study also suggests that ROS levels, mitochondrial amount and mitochondrial membrane potential of C6 glioma CSCs were comparable to those of non-CSCs.

## Abbreviations

ABC transporter: ATP binding cassette transporter
CellROX: CellROX deep red reagent
CSC: Cancer stem cell
JC-1: 5, 5′, 6, 6′-tetrachloro-1, 1′, 3, 3′-tetraethylbenzimidazolylcarbocyanine iodide
MFI: Mean fluorescent intensity
MP: Main population
MTG: MitoTracker green
SP: Side population
